# Genetic and environmental contributions to variations on appetitive traits at 10 years of age: a twin study within the Generation XXI birth cohort

**DOI:** 10.1007/s40519-021-01322-1

**Published:** 2021-11-06

**Authors:** Sarah Warkentin, Milton Severo, Alison Fildes, Andreia Oliveira

**Affiliations:** 1 EPIUnit, Institute of Public Health, University of Porto, Rua das Taipas, 135-139, 4050-600 Porto, Portugal; 2 Laboratory for Integrative and Translational Research in Population Health (ITR), Porto, Portugal; 3 Department of Public Health and Forensic Sciences and Medical Education, Faculty of Medicine, University of Porto, Porto, Portugal; 4 School of Psychology, University of Leeds, Leeds, LS2 9JT England

**Keywords:** Twins, Children, Feeding behaviors, Appetite, Heritability, Cohort studies

## Abstract

**Purpose:**

Given the variability in adiposity despite ubiquitous exposure to obesogenic food environments, it has been suggested that individuals respond in divergent ways to the environment they live in. The food environment becomes more ‘permissive’ as children age; therefore, genetic predisposition for a more avid appetite can be better expressed, influencing dietary quality, energy intake and weight gain. Our aim was to explore the genetic and environmental contribution of variations on appetitive traits in a sample of 10-year-old Portuguese children.

**Methods:**

Participants were twins enrolled in the Generation XXI birth cohort (*n* = 86 pairs). Parents reported twin’s zygosity and child appetitive traits at 10 years of age through the Children’s Eating Behavior Questionnaire. Intra-class correlations (ICCs) for all appetitive traits were calculated for monozygotic and dizygotic twins separately to examine patterns of resemblance, and structural equation modeling was conducted aiming to estimate the genetic (*A*), shared (*C*) and non-shared (*E*) environmental variances.

**Results:**

Moderate to strong heritability were found for child appetitive traits, with higher ICCs among monozygotic twin pairs. For all appetitive traits, with the exception of emotional undereating, genetic and non-shared environmental effects contributed to appetite variability. For emotional undereating, environmental effects seem to be more important than genetic effects (*C*: 0.81; 95% CI 0.71; 0.88 and *E*: 0.19; 95% CI 0.12; 0.29).

**Conclusion:**

There was a significant genetic contribution, followed by non-shared environmental contribution, towards variation in appetitive traits in school-age children. Variation in emotional undereating was primarily explained by shared and non-shared environmental factors.

**Level of evidence:**

Evidence obtained from well-designed cohort or case–control analytic studies.

**Supplementary Information:**

The online version contains supplementary material available at 10.1007/s40519-021-01322-1.

## Introduction

Genetic and environmental factors interact in many complex ways in the development of childhood obesity [1]. Given the great individual variability in adiposity, and the increasing exposure to modern day obesogenic food environments over time, it is believed that individuals respond in divergent ways to the (food) environment that they live in, i.e., genetic predispositions to being overweight seem to be enhanced in higher risk home environments in preschoolers [2] and tend to increase from early childhood to school-age years [3] until late adolescence [4], reflecting the greater external exposure to obesogenic food environments [5], which can be defined as a genotype–environment interaction [6]. A large international study with over 12,000 twin pairs, reported that heritability of body mass index (BMI) increased substantially from approximately 8% at birth, to at least 50% at 5 years and around 75% in middle-childhood (7–9 years of age). In contrast, common environmental influences, i.e., those shared by twins in the same family, were higher at birth (approximately 75%) and markedly reduced over time (9.6% at 8 years and 0% at 16 years) [7]. Increases in BMI heritability were also described in another large study with data from 45 cohorts across the world, in which genes accounted for approximately 41% in BMI variability at 4 years and increased to 75% at 19 years, in both sexes. On the other hand, a strong influence of common environmental factors was observed in middle-childhood and disappeared after 15 years of age [8].

The gene–environment interplay in the development of obesity led researchers to the behavioral susceptibility theory (BST) of obesity [9], which proposes that genetic risk operates through inherited appetitive traits that confer individual’s differential susceptibility to the food environment [5]. BST proposes a gene–environment interaction in which individuals with greater obesogenic appetitive traits, such as higher food responsiveness and lower satiety responsiveness, are more likely to overeat in situations when high palatable foods are available [5, 9]. This idea has been supported by twin studies, which have shown substantial genetic influence on potentially obesogenic appetitive traits in children, such as food responsiveness and enjoyment of food [10], eating rate [11] and eating in the absence of hunger [12], with modest effects of shared and non-shared environment. Genes seem to explain somewhere between ~ 46 and 74% of child appetitive traits (such as slowness in eating, satiety responsiveness [13] and food fussiness [14]) in infants and toddlers, ~ 72–78% of food fussiness [15] and food neophobia [16] in preschool- and ~ 63–75% of satiety responsiveness/slowness in eating and enjoyment of food [10] in school-age years, also indicating an important genetic contribution in appetite variability across childhood. In contrast, other appetitive traits, such as emotional over- and undereating, seem to be less explained by genes, as shown in a study with 5-year-old twins, in which environmental cues seemed to strongly contribute to the variation of these traits [17].

When the child ages, parental influence tends to decrease and children gain autonomy concerning their food choices. Therefore, genetic predisposition for a more avid appetite can be better expressed, increasing the power for both genetic and environmental factors to influence energy intake and ultimately weight gain [5]. In accordance with the heritability estimates for BMI, as children age and gain autonomy, you may also expect increases in the genetic contribution to certain appetitive traits [17]. However, a longitudinal study in Canada suggested that, as children age, genetic influences on appetitive traits tend to decrease [18]. It is also worth mentioning that eating behaviors are also influenced by the cultural background, which plays a role on what is eaten, when, how much and with whom [19]. Further twin studies are, therefore, necessary to better comprehend genetic and environmental contributions to a range of appetitive traits at different stages of childhood, and also from different cultural backgrounds.

The majority of twin studies assessing genetic and environmental variability in child appetitive traits are from the UK [10, 11, 14, 17] and a limited number of studies have included children in school-aged years [10, 11]. To expand on previous findings in an older age-group, aiming to test if heritability of appetitive traits increases with child autonomy, in a different culture, with different eating environments, we aimed to explore genetic and environmental contributions to variation in appetitive traits among 10-year-old children from a Portuguese population-based birth cohort. In line with previous studies, we predicted that (i) appetitive traits in 10-year-old children would be highly heritable and (ii) environmental factors may be important underlying factors in child’s emotional eating.

## Materials and methods

### Study population

Participants were children enrolled in the Generation XXI birth cohort. Recruitment took place between April 2005 and August 2006, in all public maternities of the metropolitan area of Porto (northern Portugal). Demographic and socioeconomic characteristics, obstetric history and previous personal diseases were collected in the maternity, within 72 h after delivery, in face-to-face interviews performed by trained interviewers and from clinical records [20, 21]. After the initial evaluation at birth (8647 children and 8495 mothers), follow-ups were conducted when children were 4 (2009/2011) (86% of participation proportion), 7 (2012/2014) (80% of participation proportion) and 10 years of age (2015/2017) (76% of participation proportion). From the 8647 children enrolled at baseline, the current study included only twin pairs (*n* = 288). Children with no information about zygosity (*n* = 36) and without data on eating behaviors at 10 years (*n* = 80) were excluded, resulting in a final sample of 172 children (86 twin pairs).

### Measures

Zygosity was assessed through a nine-item questionnaire [22]. This questionnaire was completed by the mothers, and assessed their opinions about children’s zygosity (e.g., “Do you think your twins are identical (monozygotic)?”), global similarity and twin confusion (e.g., “Are the children as alike as two peas in a pod?”) and specific similarity (e.g., “Are there differences in your twins’ eye colors?”). In addition, zygosity assessment was also performed by an independent observer, which classified the children as monozygotic (MZ), dizygotic (DZ) or unknown. A score of one point was given for each answer indicating similarity for a trait between twins and one point was subtracted for each answer indicating dissimilarity. A final score lower than 0 corresponded to DZ twins and scores equal to or above 0 corresponded to MZ twins. This questionnaire is a non-invasive tool of zygosity assessment and showed, previously, a high degree of accuracy (95.4%) [22].

Eating behaviors at 10 years were assessed through a widely used parent-report questionnaire, namely the Children’s Eating Behavior Questionnaire (CEBQ) [23] which was previously validated among the Generation XXI school-aged children [24]. This questionnaire also showed good internal consistency at 10 years (Cronbach’s *α* coefficients ranging from 0.76 to 0.84 [25]). Parents or main caregivers were asked to respond to the 35-item questionnaire, which assesses eight subscales: satiety responsiveness (CEBQ-SR—5 items, e.g., “My child leaves food on his/her plate at the end of a meal”), slowness in eating (CEBQ-SE—4 items, e.g., “My child eats slowly”), food fussiness (CEBQ-FF—6 items, e.g., “My child is difficult to please with meals”), emotional undereating (CEBQ-EUE—4 items, e.g., “My child eats less when s/he is upset”) assess child’s avoidance and lack of interest towards foods and will, therefore, be further called “food avoidant behaviors”; food responsiveness (CEBQ-FR—5 items, e.g., “If allowed to, my child would eat too much”), enjoyment of food (CEBQ-EF—4 items, e.g., “My child loves food”), desire to drink (CEBQ-DD—3 items, e.g., “My child is always asking for a drink”) and emotional overeating (CEBQ-EOE—4 items, e.g., “My child eats more when annoyed”) assess child’s general appetite and interest for food and drinks and will, therefore, be called “food approach behaviors”. Answers were given using a 5-point Likert scale, ranging from 1—“Never” to 5—“Always”, such that the higher the score, the more frequent the eating behavior. In accordance with the original scale, five of the items were reverse-scored. For questionnaires that were missing < 50% of data items (approximately 3% of the sample), subscale scores were calculated by replacing missing items with the mean of the items available. Adequate internal consistency was also observed in the current twins’ sample, with Cronbach’s *α* coefficients ranging from 0.77 to 0.86 (data not shown). In the majority of cases, the CEBQ at the 10-year follow-up was answered by the mother of the children (92.6%).

At each follow-up, participants were weighed, by trained researchers, in underwear and without shoes, using a digital scale and the measure was recorded to the nearest 0.1 kg. Height was also measured without shoes, using a fixed stadiometer to the nearest 0.1 cm. Children’s BMI was calculated and cutoff points for the sex- and age specific BMI *z*-scores were created. Weight status was then defined as ‘underweight’ for *z*-scores below − 2 standard deviations (SD), ‘normal weight’ for *z*-scores ≥ − 2SD and ≤ 1SD, ‘overweight’ for *z*-scores > 1 and ≤ 2SD and ‘obesity’ for *z*-scores above 2SD, according to the World Health Organization (WHO) child growth references [26].

Maternal demographic characteristics, such as age, education, marital status, pre-pregnancy weight and height, gestational age, and household monthly income were obtained through face-to-face interviews conducted by trained researchers. Mother’s weight status was classified as follows: BMI < 18.5 kg/m^2^ for ‘underweight’, between ≥ 18.5 and < 25 kg/m^2^ for ‘normal weight’, between ≥ 25 and < 30 kg/m^2^ for ‘overweight’ and ≥ 30 kg/m^2^ for ‘obesity’, according to WHO cut-offs [27]. Child birth weight was recorded in grams after birth and were retrieved from medical records by trained researchers. Birth weight categories were defined as < 1000 g as ‘extremely low’, between 1000 and 1499 g as ‘very low’, between 1500 and 2499 g as ‘low’ and ≥ 2500 g as ‘normal’, according to WHO thresholds [28], and was used for descriptive purposes only.

### Statistical analysis

Continuous variables were expressed as mean and standard deviations (M(SD)) for symmetric distributed variables, or median and interquartile ranges (Md(IQR)) for non-symmetric distributed variables. Distribution of variables was evaluated for each continuous variable using Kolmogorov–Smirnov test and graphically, through *Q*–*Q* plots. Counts and percentages (*n*(%)) were described for categorical variables.

To explore genetic and environmental contributions on variations of the CEBQ subscales, intra-class correlation coefficients (ICC) calculations (two-way mixed-single measure) and the twin method [29] were used. First, to assess the resemblance between identical and fraternal twins, ICCs for each CEBQ subscale were calculated. Greater similarities (i.e., greater ICCs) between MZ twins, compared to DZ twins, indicate a greater genetic contribution to the variation of the trait, because the only difference between these two types of twins is that the identical twins (MZ) are twice as similar genetically [17].

The twin method consists of a formal comparison between the resemblance between identical (MZ) and fraternal twins (DZ) for some traits of interest. MZ twins are genetically identical (100%) and, if reared together, share the same environment, and DZ twins share half of their genes (50%) and, if reared together, also share the same environment [29]. The maximum likelihood structural equation modeling (MLSEM) was conducted aiming to estimate the genetic and environmental variances in the measured eating behaviors from the twin method. The genetic component comprises additive genetic influences (*A*). The environmental component compromises common or shared environmental influences (*C*), which represent factors common to both twins (e.g., socioeconomic status) and child-specific or non-shared environmental factors (*E*), which refer to factors in the environment that make members of the twin pair different (e.g., an illness in only one twin) [30]. Parameter estimates, their respective 95% confidence intervals (CI), as well as models’ goodness-of-fit statistics were evaluated and described. Although we tested the full ACE model for each appetitive trait, considering the small sample size and the fit index, we only reported bivariate estimates of *A*, *C* and *E* (i.e., AC, AE and CE). These models were then compared according to the following fit indexes: Bayesian information criterion (BIC) value, log likelihood ratio (− LL), Chi-squared value (*χ*^2^) and *p* value. Only models with the best fit, i.e., that explained the observed variance and covariance with the fewest parameters for each appetitive trait, were described (models with the lowest BIC value, smallest *χ*^2^ and *p* > 0.05) [31]. Details of the remaining fitted models are described in a Supplementary Table (Supplementary Table 1).

Descriptive statistics and ICCs were performed in SPSS (IBM Corp. Released 2017. IBM SPSS Statistics for Windows, Version 25.0. Armonk, NY: IBM Corp.) and the MLSEM was performed in R 3.0.1 using the structural equation modeling package NlsyLinks [32].

## Results

Demographic and anthropometric characteristics of the 172 mother–child dyads are described in Table 1. Sixty-five percent of children were DZ twins, and nearly 63% were born with low weight (< 2500 g). At 10 years, a third of twins were classified as having overweight or obesity (30.2%).

**Table 1:** Characteristics of the Generation XXI twin sample (*n* = 172 or 86 twin pairs)

**Mother**	
Age at baseline (years)—Md (IQR)	31.0 (5.0)
Education at baseline (years)—Md (IQR)	12.0 (9.0)
Household monthly income (€)—*n* (%)	
≤ 1000	52 (33.3)
1001–2000	59 (37.8)
> 2000	45 (28.8)
Marital status—*n* (%)	
Married/in a relationship	168 (97.7)
Single/separated/divorced/widowed	4 (2.3)
BMI at baseline (kg/m^2^)—Md (IQR)	23.1 (5.0)
Weight status at baseline^a^—*n* (%)	
Underweight (< 18.5 kg/m^2^)	8 (4.8)
Normal weight (18.5–24.9 kg/m^2^)	111 (66.1)
Overweight (25.0–29.9 kg/m^2^)	33 (19.6)
Obesity (≥ 30.0 kg/m^2^)	16 (9.5)
**Child**	
Sex—*n* (%)	
Male	89 (51.7)
Female	83 (48.3)
Zygosity—*n* (%)	
MZ	60 (34.9)
DZ	112 (65.1)
Gestational age (weeks)—Md (IQR)	36.0 (3.0)
Birth weight (g)—Md (IQR)	2302.5 (754)
Birth weight^b^—*n* (%)	
Extremely low (< 1000 g)	4 (2.3)
Very low (1000–1499 g)	22 (12.8)
Low (1500–2499 g)	82 (47.7)
Normal (≥ 2500 g)	64 (37.2)
Age (years)—Md (IQR)	10.0 (0.0)
BMI *z*-score—M (SD)	0.2 (1.3)
Weight status^c^—*n* (%)	
Underweight (< − 2SD)	7 (4.0)
Normal weight (≥ − 2SD and ≤ 1SD)	113 (65.7)
Overweight (> 1 and ≤ 2SD)	33 (19.2)
Obesity (> 2SD)	19 (11.0)

*M* mean, *SD* standard deviations, *Md* median, *IQR* interquartile range, *MZ* monozygotic twins, *DZ* dizygotic twins, *BMI* body mass index

^a^Mother weight status categories were defined according to the WHO cut-offs [27]

^b^Birth weight categories were defined according to the WHO cut-offs [28]

^c^Child weight status categories were defined according to the WHO child growth references [26]

CEBQ subscales are described and ICCs for MZ and DZ twin pairs are shown in Table 2. Means of appetitive traits did not differ between MZ and DZ twins. For all appetitive traits, ICCs for MZ twins were significant and higher, compared to DZ twins. However, 95% CI overlapped for all four food approach behaviors (i.e., enjoyment of food, food responsiveness, desire to drink and emotional overeating) and for emotional undereating. For the latter, a significantly higher ICC was observed among DZ twins (ICC: 0.76; 95% CI 0.62; 0.85), suggesting that this trait has strong environmental factors explaining its variability. ICCs were significantly higher in MZ twins, compared to DZ twins, with non-overlapping 95% CI, for satiety responsiveness, slowness in eating and food fussiness.

**Table 2:** Appetitive traits descriptive, intra-class correlation coefficients (ICCs) and respective confidence intervals (95% CI), according to twin’s zygosity, at 10 years of age (*n* = 86 twin pairs)

Child appetitive traits (CEBQ)	Zygosity	
	MZ	DZ
	ICC (95% CI) M (SD)	
Enjoyment of food	**0.81 (0.62; 0.90)**	**0.56 (0.35; 0.72)**
	3.10 (0.72)	3.16 (0.77)
Food responsiveness	**0.68 (0.41; 0.83)**	**0.47 (0.24; 0.65)**
	2.12 (0.68)	2.05 (0.73)
Desire to drink	**0.86 (0.72; 0.93)**	**0.57 (0.37; 0.73)**
	1.99 (0.53)	2.09 (0.66)
Emotional overeating	**0.88 (0.75; 0.94)**	**0.63 (0.44; 0.77)**
	1.89 (0.62)	1.84 (0.62)
Satiety responsiveness	**0.88 (0.77; 0.94)**	**0.37 (0.13; 0.58)**
	2.61 (0.63)	2.56 (0.65)
Slowness in eating	**0.76 (0.55; 0.88)**	0.04 (− 0.22; 0.29)
	2.68 (0.90)	2.76 (0.89)
Food fussiness	**0.71 (0.48; 0.85)**	0.10 (− 0.16; 0.35)
	2.86 (0.66)	2.94 (0.79)
Emotional undereating	**0.89 (0.78; 0.95)**	**0.76 (0.62; 0.85)**
	2.28 (0.79)	2.33 (0.68)

Bold values are statistically significant (*p* ≤ 0.05)

*M* means, *SD* standard deviations, *MZ* monozygotic twins, *DZ* dizygotic twins, *ICC* intra-class correlations, *CEBQ* Children’s Eating Behavior Questionnaire

Genetic and environmental parameter estimates and goodness-of-fit statistics for the best-fitted model, for each appetitive trait at 10 years, are described in Table 3. For all appetitive traits, with exception of emotional undereating (CEBQ-EUE), the best model was estimated by additive genetic (*A*) and non-shared environmental (*E*) effects. Genetic effects were stronger for enjoyment of food (82%), desire to drink (90%), emotional overeating (87%) and satiety responsiveness (88%). For emotional undereating, environmental effects seem to be more important than genetic effects (shared environment—*C*: 0.81; 95% CI 0.71; 0.88 and non-shared environment—*E*: 0.19; 95% CI 0.12; 0.29).

**Table 3:** Genetic and environmental effects on variations of the CEBQ subscales (best fitted models, parameter estimates and respective confidence intervals (95% CI) at 10 years of age (*n* = 86 twin pairs)

	Additive genetic effect (*A*)	Shared environment effect (*C*)	Non-shared environment effect (*E*)	df	BIC	*χ*^2^	− LL	∆ − LL	*p* value
Enjoyment of food									
AE	0.82 (0.68; 0.93)	–	0.18 (0.07; 0.32)	7.00	356.327	11.299	− 171.464	5.649	0.126
Food responsiveness									
AE	0.69 (0.41; 0.93)	–	0.31 (0.07; 0.59)	7.00	363.020	11.170	− 174.794	5.585	0.131
Desire to drink									
AE	0.90 (0.78; 0.97)	–	0.10 (0.04; 0.23)	7.00	281.631	7.312	− 134.100	3.656	0.397
Emotional overeating									
AE	0.87 (0.78; 0.95)	–	0.13 (0.05; 0.22)	7.00	271.734	8.854	− 129.134	4.427	0.263
Satiety responsiveness									
AE	0.88 (0.77; 0.95)	–	0.12 (0.05; 0.23)	7.00	304.246	4.310	− 145.407	2.155	0.743
Slowness in eating									
AE	0.69 (0.19; 0.94)	–	0.31 (0.06; 0.81)	7.00	448.635	10.394	− 217.618	5.197	0.167
Food fussiness									
AE	0.70 (0.08; 0.95)	–	0.30 (0.05; 0.93)	7.00	396.215	13.071	− 191.391	6.535	0.070
Emotional undereating									
CE	–	0.81 (0.71; 0.88)	0.19 (0.12; 0.29)	7.00	298.774	5.275	− 142.671	2.638	0.626

*CEBQ* Children’s Eating Behavior Questionnaire, *A* additive genetic component of variance, *C* shared environmental component of variance, *E* non-shared environmental component of variance, *df* degrees of freedom, *BIC* Bayesian information criterion, *χ2* Chi-squared test,* − LL* log-likelihood of data

## Discussion

Several appetitive traits at 10 years showed moderate to high heritability, but they were also partially explained by the individual’s unique environment (i.e., the non-shared environment). On the other hand, children’s tendency to eat less in response to negative emotions seem to be determined by the shared and non-shared environments, rather than being genetically predicted.

The heritability estimates observed for the food approach behaviors are in line with previous studies [10, 11, 18]. Specifically, among Canadian children, heritability of eating too much and eating too fast in 2.5 years was estimated to be 87% and 71%, respectively, and, in the 9-year-old sample, eating between meals showed 72% of genetic predisposition [18]. In the UK, food responsiveness was estimated to be 75% heritable in 8–11 years [10], and heritability of eating rate, among 10–12 years, was estimated to be 62% [11]. These findings indicate that being reared in the same household has a relatively small effect on making twins similar to one another for most appetitive traits in middle-childhood [10]. The found contribution of both genetic and non-shared environmental influences on children’s appetitive traits corroborates with other studies in this same age-group [10, 11, 18], indicating that siblings have unique experiences inside and outside the household and, therefore, differ in their behavioral responses to that environment (i.e., appetitive traits). There is a general trend for no shared environmental component (i.e., the ‘C’ estimate) in specific behaviors, such as eating rate and quantity of food eaten among preschool- and school-aged children [18] and uncontrolled eating among adults [33], with the remaining non‐genetic variance being explained mostly by environmental factors that are not shared by twins (i.e., the ‘E’ estimate), which measure “each twin’s unique experiences of the world”.

A significant genetic component was also found for the food avoidant behaviors. The genetic component underlying food fussiness, for example, has been described to be 46% among 16-month-old children [14], 78% among 3 years [15], 73% among 9 years (assessed by a single question about the child being fussy about eating vegetables) [18] and 70% in the current sample, which suggests that this appetitive trait seems to increase its genetic predisposition in the first years of life. In addition to the genetic effect, children’s non-shared/unique environment also seemed to explain more in the variability of food fussiness, compared to the shared environment, and this corroborates with previous studies in preschool- [15] and in school-age years [18]. These findings suggest that the home environment shapes this particular trait especially in early life, being, therefore, a sensitive period to intervene. The early life home environment includes a variety of aspects, such as the availability and accessibility to healthy foods, which have shown to be associated with child greater food consumption and less food fussiness [34], as well as the use of specific parent-feeding strategies, such as repeated exposure to fruit and vegetables [35], which decreases fussy eating. In addition, parents may act as role models for a healthier food consumption and providers of a mealtime structure [36], which have shown to have positive consequences on food preferences and dietary quality, decreasing fussy eating, and should, therefore, be encouraged in early years.

Our second hypothesis was partially confirmed. We found that emotional undereating was largely explained by environmental factors (shared and non-shared), with genetic influences playing a minor role; however, emotional overeating showed an important genetic predisposition in our sample (‘A’: 0.87, 95% CI 0.78; 0.95). A study that analyzed data from the Gemini twin study in the UK found, besides the great effect of environmental factors on emotional undereating, that emotional overeating was also largely explained by environmental factors [17]. The main differences to our study are the English younger age-group (5 years of age) and larger sample size (*n* = 1027 twin pairs), so heritability may have increased in this appetitive trait among the older children in our study or we may have lacked in power to estimate environmental effects on emotional overeating. However, twin studies among adults have shown effects of both genetic and non-shared environmental factors on emotional overeating [37, 38], suggesting that genetic influences on this trait may also increase over time, as seen with other eating behaviors. As described in a review by Herle and colleagues [39], emotional overeating is affected by genetic and environmental cues that vary considerably throughout life. This behavior emerges in early ages, and its etiology may change when the child ages. The home environment (i.e., parental influence), for example, seems to play a major role in its development among young children, and external factors, such as peers, show a significant importance later in childhood. However, further studies are necessary to confirm this hypothesis.

A variety of environmental factors may influence child’s emotional eating; however, the specific environmental cues that influence children’s appetitive traits were not investigated in the present study. Four levels of environmental factors that may influence eating behaviors have been suggested by Story et al., as follows: individual factors (which include psychological and biological factors), the social environment (such as parents and peers), the physical environment (such as schools, restaurants and food availability) and the societal level in which children live in (which include, for example, cultural and social norms, marketing and advertising) [40]. The primary influence on child eating behaviors often occur at home, particularly with parents engaging in feeding practices [30] to maintain or modify child’s weight and/or eating habits [41]. However, corroborating with our results (with exception to emotional undereating), others found small effects of twin’s shared environment on appetitive traits [15, 18] and BMI in childhood [42]. Even though siblings experience the same food environment, are served with similar foods, see the same behaviors modeled by parents, have similar indoor and outdoor activities, and go to the same school, these results show that these factors do not make siblings more similar—which is a challenge for obesity etiological models that highlight the home environment as a root for obesity development [42].

The effect of genes underlying pediatric appetitive traits does not mean that environmental changes to improve dietary quality and/or prevent inadequate weight do not work, but rather that this control is likely to be more difficult for some children, given the combination of genetically determined appetite and the obesogenic food environment that increases opportunities to overeat [10]. This means that, as vastly described, the food environment has a great influence on eating behaviors and weight gain in childhood [2, 43, 44] and adolescence [40, 45], being, therefore, public health interventions highly encouraged when necessary.

## Strength and limits

This study has some limitations that need to be addressed. First, a major limitation is the small sample size of twin pairs in the Generation XXI birth cohort, which may have decreased the reliability of the parameter estimates and widened and overlapped confidence intervals. A larger sample size might have allowed detecting smaller additive and environmental influences. The wide confidence intervals for the parameter estimates indicate the need for replication in larger samples. It was not possible to carry out a power analyses prior to this cohort study. However, post hoc analyses revealed the study sample of 172 children had approximately 40% power to detect a significant genetic effect with a variance component of 0.33 and a shared environment variance component of 0.1 [46]. Previous twin studies conducted in the UK used large samples of twins [13, 14, 17], which led to more precise estimations of genetic and environmental contributions on appetitive traits. A strength of ours study is that twins from an ongoing prospective cohort were studied, instead of a sample of twins specifically recruited to answer this objective, which brings several advantages from the multiple and extensive follow-up conducted across time.

Second, the parent-report nature of children’s appetitive traits may have introduced measurement error due to subjectivity and social desirability bias [47]. In addition, it represents parent’s perceptions and child eating behaviors outside parents’ view, such as in the school environment, could be different and not described here. Another limitation is the evaluation of eating behaviors using a common source or rater (in this case, the mother responded for both twins). As described in the review by Podsakoff et al., people try to appear consistent and rational in their answers, producing relationships that would not appear in real-life settings [47]. However, the CEBQ has demonstrated good internal consistency in this population [24] and good correspondence with objective behavioral measures of children’s eating [48]. In addition, a limitation of the twin method is the equal-environment assumption, which could overestimate heritability estimates. It has been discussed by others [49, 50] that the assumption of equal shared environment among MZ and DZ may not be entirely true, arguing that MZ twins experience environments that are more similar compared to DZ twins. In the comprehensive review of Felson [50], the use of equal-environment assumption in twin studies is valid in most cases; however, violations of this assumptions may also exist and increase heritability estimates. In twin studies assessing eating behaviors and attitudes, the equal-environment assumption has shown to be reliable [51].

This is, to our knowledge, the first study among Portuguese children that assessed heritability of appetitive traits, which extend the previous findings from other populations. The findings of this study suggest that there is a significant genetic contribution for the establishment of appetitive traits among 10 years, especially for enjoyment of food (82%), desire to drink (90%), emotional overeating (87%) and satiety responsiveness (88%). Despite the great heritability of appetitive traits in school-age years, variability of behaviors was also explained by the unique environment, which can be addressed in public health interventions. Emotional undereating was the only trait that was not heritable, with a significant proportion of this trait being explained by both common and unique environmental factors. The recognized and complex genotype–environment interaction needs to be considered when investigating the etiology of appetitive traits. Understanding which genes are associated with appetitive traits in childhood would give a powerful insight in the biological and behavioral influences on child eating and obesity risk [30].

## What is already known on this subject?

There is a complex gene–environment interplay in the development of obesity, which proposes that its genetic risk operates through inherited appetitive traits that confer individual’s differential susceptibility to the food environment. Twin studies in the UK have shown substantial genetic influence on potentially obesogenic appetitive traits in children.

## What your study adds?

This study expands on previous findings in a different culture and older age-group on the genetic and environmental variability in appetitive traits in a Portuguese sample of school-age children.

## Supplementary Information

Below is the link to the electronic supplementary material.

Supplementary file1 (DOCX 16 kb)

## Data Availability

The data sets generated during and/or analysed during the current study are available from the corresponding author on reasonable request.
