# Supplementary material for: Genetic and environmental contributions to variations on appetitive traits at 10 years of age: a twin study within the Generation XXI birth cohort
Source: Eat Weight Disord. 2021 Nov 6;27(5):1799–807. doi: 10.1007/s40519-021-01322-1 (PMC9123061; doi:10.1007/s40519-021-01322-1)
Supplement: Supplementary file 1 — Supplementary file1 (DOCX 16 kb) [file 40519_2021_1322_MOESM1_ESM.docx]

**Supplementary Table 1.** Model fit of remaining submodels for appetitive traits at 10 years of age.

|  | **df** | **BIC** | **χ^2^** | **-LL** | **∆-LL** | **p-value** |
| --- | --- | --- | --- | --- | --- | --- |
| **Enjoyment of Food** |  |  |  |  |  |  |
| ACE | 6.00 | 358.799 | 9.306 | -170.468 | 4.653 | 0.157 |
| CE | 7.00 | 361.505 | 16.477 | -174.054 | 8.239 | 0.021 |
| E | 8.00 | 401.624 | 61.063 | -196.346 | 30.531 | 0.000 |
| **Food Responsiveness** |  |  |  |  |  |  |
| ACE | 6.00 | 367.213 | 10.886 | -174.652 | 5.443 | 0.092 |
| CE | 7.00 | 366.630 | 14.781 | -176.599 | 7.39 | 0.039 |
| E | 8.00 | 386.294 | 38.921 | -188.669 | 19.46 | 0.000 |
|  |  |  |  |  |  |  |
| **Desire to Drink** |  |  |  |  |  |  |
| ACE | 6.00 | 284.989 | 6.192 | -133.540 | 3.096 | 0.402 |
| CE | 7.00 | 297.446 | 23.127 | -142.007 | 11.563 | 0.002 |
| E | 8.00 | 339.959 | 70.117 | -165.502 | 35.058 | 0.000 |
| **Emotional Overeating** |  |  |  |  |  |  |
| ACE | 6.00 | 271.941 | 4.572 | -126.993 | 2.286 | 0.600 |
| CE | 7.00 | 277.681 | 14.801 | -132.107 | 7.400 | 0.039 |
| E | 8.00 | 336.025 | 77.634 | -163.524 | 38.817 | 0.000 |
| **Satiety Responsiveness** |  |  |  |  |  |  |
| ACE | 6.00 | 308.723 | 4.310 | -145.407 | 2.155 | 0.635 |
| CE | 7.00 | 327.015 | 27.079 | -156.792 | 13.540 | 0.000 |
| E | 8.00 | 350.629 | 55.170 | -170.837 | 27.585 | 0.000 |
| **Slowness in Eating** |  |  |  |  |  |  |
| ACE | 6.00 | 453.100 | 10.394 | -217.618 | 5.197 | 0.109 |
| CE | 7.00 | 459.402 | 21.162 | -223.002 | 10.581 | 0.004 |
| E | 8.00 | 462.194 | 28.420 | -226.631 | 14.210 | 0.000 |
| **Food Fussiness** |  |  |  |  |  |  |
| ACE | 6.00 | 400.692 | 13.071 | -191.391 | 6.535 | 0.042 |
| CE | 7.00 | 405.183 | 22.039 | -195.876 | 11.020 | 0.003 |
| E | 8.00 | 405.352 | 26.685 | -198.199 | 13.343 | 0.001 |
|  |  |  |  |  |  |  |
| **Emotional Undereating** |  |  |  |  |  |  |
| ACE | 6.00 | 301.063 | 3.087 | -141.577 | 1.544 | 0.798 |
| AE | 7.00 | 314.558 | 21.060 | -150.563 | 10.530 | 0.004 |
| E | 8.00 | 388.197 | 99.175 | -189.621 | 49.588 | 0.000 |

A, Additive genetic component of variance; C, Shared environmental component of variance; E, Non-shared environmental component of variance; df, Degrees of freedom; BIC, Bayesian information criterion; χ2, Chi-squared test; -LL, Log-likelihood of data.
